# Psychometric evaluation of the 8-item Altarum Consumer Engagement (ACE) Measure™ in community-dwelling adults in Singapore

**DOI:** 10.1186/s12913-021-07369-1

**Published:** 2021-12-16

**Authors:** Lixia Ge, Chun Wei Yap, Palvinder Kaur, Reuben Ong, Bee Hoon Heng

**Affiliations:** Health Services and Outcomes Research, National Healthcare Group, 3 Fusionopolis Link #03-08, Nexus@one-north, Singapore, 138543 Singapore

**Keywords:** Engagement, Psychometric properties, Validation, Commitment, Activation

## Abstract

**Background:**

A valid and reliable measure is essential to assess patient engagement and its impact on health outcomes. This study aimed to examine the psychometric properties of the 8-item Altarum Consumer Engagement Measure™ (ACE Measure) among English-speaking community-dwelling adults in Singapore.

**Methods:**

This cross-sectional study involved 400 randomly selected community-dwelling adults (mean age: 49.7 years, 50.0% were female, 72.3% were Chinese) who completed the English version of the 8-item ACE Measure independently. The item-level statistics were described. The internal consistency of the measure was measured by Cronbach alpha and item-rest correlations. Validity of the tool was assessed by 1) factorial validity using confirmatory factor analysis (CFA), 2) hypothesis-testing validity by correlating ACE subscales (Commitment and Navigation) with health-related outcomes, and 3) criterion validity against the Patient Activation Measure and Health Confidence Measure.

**Results:**

There was no floor or ceiling effect for Commitment and Navigation subscales, and the Cronbach alpha for each subscale was 0.76 and 0.54, respectively. The two-factor structure was confirmed by CFA. In general, Commitment and Navigation subscales were positively correlated with frequency of activity participation (rho = 0.30 - 0.33) and EQ-5D visual analog scale (rho = 0.15 - 0.30). Individuals who perceived better health than peers had higher subscale scores (*p* < 0.01). Each subscale score had moderate and positive correlations with activation score (rho = 0.48 - 0.55) and health confidence score (rho = 0.35 - 0.47).

**Conclusions:**

The two-subscale ACE Measure demonstrated good construct validity in English-speaking Singapore community-dwelling adults. Evidence in internal consistency was mixed, indicating further investigation.

## Background

With the shift from a disease-centered to patient-centered healthcare model that focuses on disease prevention and sustained management of chronic conditions management [1, 2], healthcare systems and providers are increasingly recognizing the importance of engaging healthcare consumers in their treatment decision making process and empowering them to take on more active roles in their own health and healthcare management.

Used interchangeably, patient or health engagement is increasingly recognized as a critical component of patient-centered care. Yet it remains a poorly understood concept [3, 4] with inconsistent definitions and operationalization. Prior literature has suggested that patient engagement is a multidimensional concept – where individuals are not only required to have the knowledge, skills, ability, and willingness to manage their own health, but also to be actively involved in making competent and well-informed decisions together with their healthcare providers and commit to take actions for own health care [3, 5, 6]. A growing body of evidence demonstrated that individuals who are engaged in their health care tend to express a stronger motivation in self-management and are better able to make informed decisions about their care options, which contributes to improved adherence [7–9], better self-perceived health [10, 11] and health outcomes (i.e. fewer depressive symptom and better physical function) [8, 12, 13], and lower healthcare costs [14, 15].

Measuring engagement in health care using a valid and reliable measure is essential to evaluate its impact on health outcomes and healthcare utilization. A few instruments have been developed in recent years to measure different domains of patient engagement including patient activation [16], motivation [17], self-management [18–20], and shared decision making [21]. The 13-item Patient Activation Measure (PAM-13) is a powerful tool that is widely used to assess an individual’s self-perceived knowledge, skills and confidence to manage one’s own health and healthcare requirements [16, 22]. The Patient Health Engagement (PHE) scale, which was developed based on the PHE model, aims to grasp the complexity and dynamicity of the psychological experience of engagement [9]. However, there is still a lack of concrete instruments which encompass the full range of dimensions of patient engagement [9, 23]. Recently, Duke and colleagues developed the Altarum Consumer Engagement Measure™ (ACE Measure) [23] to address the multi-dimensionality of health engagement.

The ACE Measure was developed to assess the health engagement of individuals and population from multiple aspects of patient perceptions, activities of participation in health and healthcare, and the use of information to compare and choose providers or services [23]. It is not only meant for disease management, but also suitable for population health management. The original 21-item ACE Measure is a multi-dimensional health engagement measure which consists of four subscales: Commitment - confidence and ability to maintain a healthy lifestyle and manage one’s health, Ownership - perceived role in and responsibility for one’s health, Informed Choice - patterns of seeking and using information about health and healthcare, and Navigation - confidence and ability to ask about and participate in treatment decisions [23]. It was subsequently shortened to 12 items consisting of three subscales: Commitment (4 items), Informed Choice (4 items), and Navigation (4 items). The Ownership subscale was not included due to its similarity in outcome prediction with that of the Commitment subscale [24].

The 12-item ACE Measure has been validated among United States prediabetes patients [24] and has been used in many ways including population benchmarking, generating patient’s personalized engagement reports, triaging resources, determining course of treatment and health coaching since its development [25]. Each subscale of the 12-item ACE Measure can be used independently. In a recent study conducted by the United States Air Force, diabetes patients with high Commitment achieved better improvement in hemoglobin A1c over 8 months [26]. However, the ACE measure has never been validated among Singapore adult population. Hence, the aim of the study was to examine the psychometric properties of the ACE Measure among English-speaking community-dwelling adults in Singapore.

## Methods

### Study participants

Data of the study was collected in the second-year follow-up survey of the Population Health Index (PHI) study via surveyor-administered face-to-face interviews at participants’ homes or places preferred by participants. The PHI survey was a longitudinal health survey conducted among community-dwelling adult population (aged 21 years and above) living in the Central region of Singapore. The sampling methods and procedures were described elsewhere [27, 28]. A total of 1942 individuals participated in the survey and they were followed up at one-year and two-year after the first survey. During the second-year follow-up survey, the English-speaking participants who were capable to complete the questionnaire independently (*n* = 978) were also invited to complete the English version ACE Measure, the PAM-13, and the Health Confidence Measure. Individuals who had invalid responses to the PAM-13 (including those with less than 10 questions answered and those with “3 = agree” responses for all 13 items) were excluded from the analysis (*n* = 152). As suggested by Nicholas and colleagues [29], a sample size of 300 or more are necessary for a confirmatory factor analysis (CFA). Hence, we randomly selected 400 participants from the remaining 826 participants for the analysis of the study.

The study obtained ethics approval from Singapore’s National Healthcare Group Domain Specific Review Board (Reference Number: 2015/00269) and was conducted in accordance with the Declaration of Helsinki. Written informed consent was obtained from each participant after being informed of the study purpose, procedures, and confidentiality of the data collected.

### Measures

#### The 8-item Altarum consumer engagement measure™

The three subscales of the 12-item ACE Measure and their respective items were listed in Table 1. The Informed Choice subscale of the 12-item ACE Measure contains two items related to official ratings of doctors (“Q8. I compare doctors using official ratings about how well their patients are doing.” and “Q10. When choosing a new doctor, I look for official ratings based on patient health.”). Unlike the US healthcare system, there are no official ratings of healthcare providers in Singapore. As such, these two items are not relevant to Singapore’s context. Upon discussion with developers of the tool, these two items were excluded from the survey. The wording of the remaining 10 items was reviewed by two authors for understandability in Singapore. As both authors felt the wording was commonly used and easy to be understood by Singapore English-speaking population, all 10 items were included in the survey without any changes. The general feedbacks obtained via the trained surveyors from the first 30 participants indicated that the items were well understood by the study population.

**Table 1:** The three subscales of the 12-item ACE Measure and the 8-item ACE Measure included for validation

Subscale	12-item ACE Measure	8-item ACE Measure	Item
Informed Choice	Q1	Excluded	I spend a lot of time learning about health.
Commitment	Q2	ACE8_1	Even when life is stressful, I know I can continue to do the things that keep me healthy.
Navigation	Q3	ACE8_2	I feel comfortable talking to my doctor about my health.
Commitment	Q4	ACE8_3	When I work to improve my health, I succeed.
Navigation	Q5	ACE8_4	I have brought my own information about my health to show my doctor.
Informed Choice	Q6	Excluded	When choosing a new doctor, I look for information online.
Commitment	Q7	ACE8_5	I can stick with plans to exercise and eat a healthy diet.
Informed Choice	Q8	Excluded	I compare doctors using official ratings about how well their patients are doing.
Navigation	Q9	ACE8_6	I have lots of experience using the health care system.
Informed Choice	Q10	Excluded	When choosing a new doctor, I look for official ratings based on patient health.
Navigation	Q11	ACE8_7	Different doctors give different advice; it’s up to me to choose what’s right for me.
Commitment	Q12	ACE8_8	I handle my health well.

Response options for each item: 0 = strongly disagree, 1 = disagree, 2 = neither agree nor disagree, 3 = agree, and 4 = strongly agree

With the removal of the two items, the Informed Choice subscale was left with two items that would cause identification problem in confirmatory factor analysis as the minimum number of items for a factor was not reached. After consulting the developer of the ACE Measure and considering that each subscale could be used independently, we only examined the psychometric properties of the two subscales - Commitment and Navigation of the 8-item ACE Measure in this study.

The 8 items of the ACE Measure used the same rating scale with 5 response options (0 = strongly disagree, 1 = disagree, 2 = neither agree nor disagree, 3 = agree, and 4 = strongly agree). Following the scoring procedure for the 21-item ACE Measure, the mean score of items in each subscale was multiplied by 6.25 to create a subscale score ranging from 0 to 25, with a higher score representing greater commitment or better navigation.

#### Health-related outcomes for hypothesis-testing validity

Frequency of activity participation and health-related quality of life (HRQoL) were the two health-related outcomes selected to examine the hypothesis-testing validity of the two subscales of the 8-item Measure. The Frequency domain of the Late Life Function and Disability [30] was used to measure frequency of activity participation and the EQ-5D-5L [31] (including EQ-5D Index and EQ-5D Visual Analog Scale (VAS)) was used to measure HRQoL. In addition, self-perceived health status was obtained by asking the question “In comparison with other people of the same age, how do you consider your health status?” with four response options (“not as good”, “does not know”, “as good”, and “better”).

#### Patient activation measure and health confidence measure for criterion validity

The 13-item Patient Activation Measure® (PAM-13) and Health Confidence Measure were chosen to examine the criterion validity of the two subscales of the 8-item ACE Measure. Developed by Hibbard and colleagues [16] using Rasch analyses, the PAM-13 is an interval-level, unidimensional and Guttman-like measure that contains 13 questions measuring self-assessed knowledge, skills and confidence for self-management of one’s health or chronic condition. PAM-13 is the most commonly used measure for patient’s activation in health and has been shown to be associated with a broad range of health-related outcomes [16, 32]. Each item of the PAM-13 has 5 response options (1 = strongly disagree, 2 = disagree, 3 = agree, and 4 = strongly agree, with additional “not applicable” option). To calculate the total PAM score, the PAM-13 scoring spreadsheet 2017 (the scoring algorithm obtained from the license provider Insignia Health) was used to calculate individual participant’s PAM-13 score ranging from 0 to 100, and to determine their activation levels. The PAM-13 scoring spreadsheet only calculated PAM score for participants with 10 or more questions answered, and participants with “3 = agree” responses for all 13 items were treated as outliers. Only participants with valid PAM-13 were sampled for this analysis.

Health Confidence Measure [20] is an effective proxy for engagement using a single question “How confident are you that you can control and manage most of your health problems?” The respondents were asked to rate their health confidence on a scale from 0 (not very confident) to 10 (very confident). A score of 7 or higher was categorized as high health confidence and a score of 6 or lower was categorized as low health confidence.

### Data analysis

#### Item-level descriptive analysis and internal consistency

Descriptive analyses for each of the 8 items were conducted as initial exploration of the data. Mean, standard deviation (SD), median and score distribution were reported for individual items. Furthermore, floor or ceiling effects were considered present if > 15% of participants achieved the lowest score / floor effect (0/4 for individual items, 0/25 for subscales) or the highest score / ceiling effect (4/4 for individual items, 25/25 for subscales) [33].

The internal consistency of each subscale of the 8-item ACE Measure was assessed by calculating Cronbach’s alpha, item-rest correlations (the correlation of the item with the total score on the other items), and average inter-item correlations (a way of measuring whether different items that are meant to measure the same general construct give similar scores). A Cronbach’s alpha of 0.70 or higher was defined as the acceptable value [34]. The ideal range of average inter-item correlation was 0.15 to 0.50 [35].

#### Factorial validity

As the 8-item ACE Measure comprises two correlated subscales - Commitment and Navigation in the 12-item ACE Measure, to examine factorial validity of the construct of the 8-item ACE Measure, two-factor CFA with the respective 4 items in each of the original subscales was conducted. Each item could load onto one latent factor with the first item’s loading onto the latent factor fixed at 1.0. For the remaining factor loadings, residual variances were freely estimated. Standardized factor loading values were calculated and multiple goodness of fit statistics of the model were performed, including the ratio of the chi-square to degrees of freedom chi-square (χ^2^/df), the Comparative Fit Index (CFI), the Tucker-Lewis Fit Index (TLI), and the Root Mean Square Error of Approximation (RMSEA). The cutoff values proposed by Hu and Bentler: χ^2^/df ≤ 3, CFI ≥0.95, TLI ≥0.95, and RMSEA ≤0.06 [36] were used to evaluate the goodness of model fit.

#### Hypothesis-testing validity

To examine the hypothesis-testing validity of the 8-item ACE Measure, the variation in the two ACE Measure subscale scores across socio-demographic subgroups were examined by Mann Whitney U tests or Kruskal-Wallis tests (followed by Dunn’s tests [37]). The difference in ACE Measure subscale scores across self-perceived health status groups was evaluated using the Kruskal-Wallis test, followed by Dunn’s tests. Furthermore, the relationships between ACE Measure scores and frequency of activity participation as well as HRQOL (measured by EQ-5D index EQ-5D VAS scores) were assessed using Spearman’s rank-order correlations. We hypothesized that 1) those with higher education levels have higher Commitment and Navigation scores; 2) those who perceived better health status than peers have higher Commitment score than their counterparts; and 3) greater engagement, especially higher commitment is associated with more frequent activity participation and higher health-related quality of life (HRQOL).

#### Criterion validity

Criterion validity of the 8-item ACE Measure was carried out against the PAM-13 and the Health Confidence Measure by examining the relationships between the two ACE subscale scores and PAM-13 and health confidence scores using Spearman’s rank-order correlations. The two ACE subscale scores were also compared across participants with different PAM activation levels and between those with high and low health confidence. We hypothesized that the two subscale scores have positive, moderate correlation with PAM-13 and health confidence scores. We also hypothesized that those with higher PAM levels and those with higher health confidence should have higher ACE subscale scores over their counterparts.

All the analyses were conducted using Stata/SE 16 for Windows. The result was considered significant if a *p* value was < 0.05.

## Results

The characteristics of the participants were described in Table 2. The average age of the 400 participants was 49.2 years (standard deviation: 15.0 years, range: 23-90 years). Half of the participants were women and 72.3% were Chinese. Majority (59.3%) of the participants had post-secondary or higher education.

**Table 2:** Participant characteristics and mean subscale scores of the 8-item ACE Measure for each subgroup (*N* = 400)

Characteristics	n	%	Commitment (Mean ± SD)	Navigation (Mean ± SD)
**All**			18.4 ± 3.2	17.2 ± 3.2
**Age group**				
21-39	112	28.0	17.9 ± 3.6	17.1 ± 3.7
40-59	181	45.3	18.5 ± 3.1	17.3 ± 2.9
60-74	90	22.5	19.1 ± 3.0**	17.6 ± 3.1
75&above	17	4.3	18.1 ± 2.5	15.8 ± 3.5
**Gender**				
Male	200	50.0	18.4 ± 3.4	17.2 ± 3.1
Female	200	50.0	18.5 ± 3.1	17.3 ± 3.4
**Ethnicity**				
Chinese	289	72.3	18.3 ± 3.3	17.2 ± 3.3
Malay	32	8.0	18.4 ± 3.2	16.9 ± 3.6
Indian	72	18.0	19.1 ± 3.1	17.5 ± 3.1
Others	7	1.8	17.6 ± 3.0	18.8 ± 0.9
**Marital status**				
Single	103	25.8	18.0 ± 3.5	16.5 ± 3.6
Married	248	62.0	18.6 ± 3.1	17.5 ± 3.0
Divorced /widowed	49	12.3	18.9 ± 3.0	17.4 ± 3.4
**Highest education attained**				
No formal education	13	3.3	17.2 ± 3.0	14.4 ± 3.2
Primary	28	7.0	17.9 ± 3.1	17.0 ± 2.3*
Secondary	122	30.5	18.3 ± 3.2	17.2 ± 3.2**
Post-secondary and above	237	59.3	18.6 ± 3.3	17.4 ± 3.3**
**Living alone**				
No	370	92.5	18.5 ± 3.2	17.3 ± 3.2
Yes	30	7.5	17.6 ± 3.8	16.2 ± 3.6
**Money sufficiency**				
Sufficient	343	85.8	18.5 ± 3.2	17.3 ± 3.2
Insufficient	57	14.3	18.0 ± 3.4	17.0 ± 3.4
**Self-perceived health**				
Not as good	26	6.5	15.1 ± 4.4	16.9 ± 3.1
Do not know	29	7.3	17.6 ± 3.6*	16.4 ± 3.3
As good	160	40.0	18.1 ± 2.7**	16.7 ± 3.2
Better	185	46.2	19.3 ± 3.0**	17.8 ± 3.2

*p < 0.05, **p < 0.01 compared to the first category in respective characteristic using Dunn’s test

### Item-level statistics and internal consistency

**Fig. 1:**
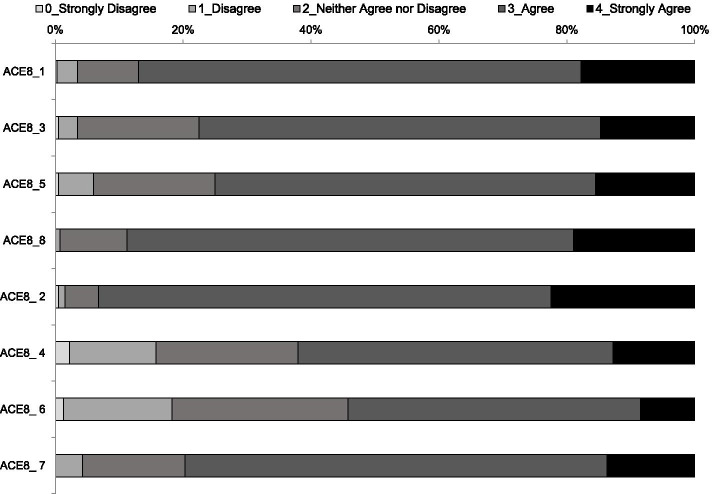
The distribution of responses by items of the two-subscale ACE Measure

Table 3 shows the descriptive statistics of individual items of the two subscales of the 8-item ACE Measure and Fig. 1 presents the distribution of the responses for each item. The means of the 4 items under Commitment and Navigation subscales were 3.0 and 2.8, respectively. Item ACE8_2 had the highest percentage of participants with response of “strongly agree” (22.5%) followed by item ACE8_8 (19.0%) and ACE8_1 (17.8%). However, the proportions of participants having the lowest or highest mean score of the 4 items under Commitment and Navigation subscales were all below 5.0%, indicating no floor or ceiling effect for each subscale.

**Table 3:** Item-level statistics, item-rest correlation, and average inter-item covariance for the two-subscale ACE Measure (*N* = 400)

Subscale	Item	Mean	SD	Median	Floor / ceiling effect (%)	Item-rest correlation	Average inter-item covariance
Commitment	ACE8_1. Even when life is stressful, I know I can continue to do the things that keep me healthy.	3.0	0.7	3	0.3 / 17.8	0.50	0.22
	ACE8_3. When I work to improve my health, I succeed.	2.9	0.7	3	0.5 / 14.8	0.61	0.18
	ACE8_5. I can stick with plans to exercise and eat a healthy diet.	2.8	0.8	3	0.5 / 15.5	0.59	0.18
	ACE8_8. I handle my health well.	3.1	0.6	3	0 / 19.0	0.56	0.23
Navigation	ACE8_2. I feel comfortable talking to my doctor about my health.	3.1	0.6	3	0.5 / 22.5	0.29	0.19
	ACE8_4. I have brought my own information about my health to show my doctor.	2.6	0.9	3	2.3 / 12.8	0.39	0.10
	ACE8_6. I have lots of experience using the health care system.	2.4	0.9	3	1.3 / 8.5	0.45	0.09
	ACE8_7. Different doctors give different advice; it’s up to me to choose what’s right for me.	2.9	0.7	3	0 / 13.8	0.21	0.21

Response options for each item: 0 = strongly disagree, 1 = disagree, 2 = neither agree nor disagree, 3 = agree, and 4 = strongly agree

The internal consistency of the 8-item ACE Measure was acceptable for Commitment subscale with a Cronbach’s alpha of 0.76. However, it was not satisfactory for Navigation subscale with a Cronbach’s alpha of 0.54. The average inter-item correlation for Commitment subscale was 0.20, with individual inter-item correlations ranging from 0.18 to 0.23, falling within the ideal range of 0.15 – 0.50. All four items for Commitment subscale had item-rest correlations ranging from 0.50 – 0.61, exceeding the cutoff score for strong correlations (≥0.50). The average inter-item correlation for Navigation subscale was 0.15, with items ACE8_4 and ACE8_6 falling below 0.15 (Table 1).

### Factorial validity

**Fig. 2:**
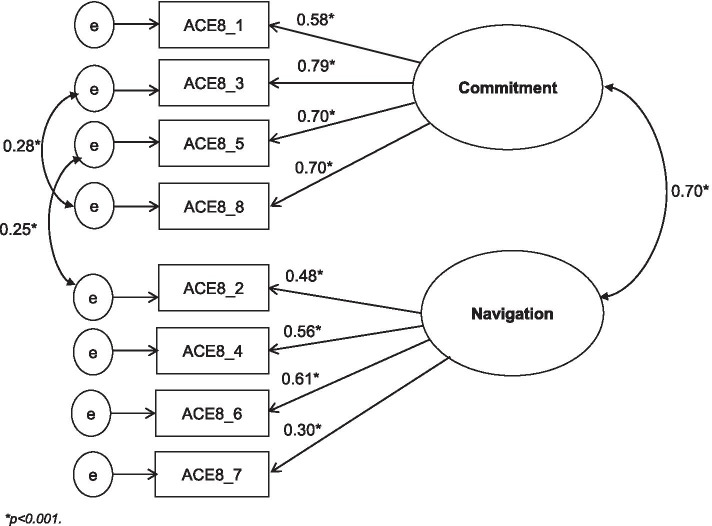
The path diagram for the two-factor CFA model: standardized estimates

The CFA confirmed the two-factor latent construct of the 8-item ACE Measure. The standardized factor loading for each item onto the respective latent construct (Commitment and Navigation) was provided in Fig. 2. All four items under Commitment subscale had a factor loading higher than 0.50. Two items under Navigation subscale had relatively lower factor loadings (0.48 for item ACE8_2 and 0.30 for item ACE8_7). The Chi-square test result (χ^2^(17) =27.43, *p* = 0.05) and goodness of fit indices (CFI = 0.98, TLI = 0.97, RMSEA = 0.04) indicated a good fit between the two-factor model and the observed data.

### Hypothesis-testing validity

As expected, there were no differences in the Commitment and Navigation scores between males and females or across ethnicity groups (Table 2). Dunn’s test showed that participants aged 60-74 years reported higher Commitment scores compared to those aged 21-39 years, and participants having higher education levels had higher Navigation scores.

Compared to participants who perceived health not as good as peers, those who perceived health as good as or better than peers had significantly higher Commitment scores (Table 2). Commitment score was positively associated with higher frequency of activity participation (rho = 0.30, *p* < 0.01) and higher EQ-5D VAS (rho = 0.30, *p* < 0.01) and Index scores (rho = 0.15, *p* < 0.01); Navigation score was associated with higher frequency of activity participation (*rho* = 0.33, *p* < 0.01) and higher EQ-5D VAS (rho = 0.15, *p* < 0.01) but not associated with higher EQ-5D Index scores (rho = 0.03, *p* = 0.570) (Table 4).

**Table 4:** Spearman correlation between two subscales of the 8-item ACE Measure and health-related outcomes (*N* = 400)

Health-related outcomes	Commitment		Navigation	
	rho	95% Confidence Interval	rho	95% Confidence Interval
Frequency of activity participation	0.30**	0.21, 0.38	0.33**	0.24, 0.41
EQ-5D VAS	0.30**	0.21, 0.39	0.15**	0.05, 0.24
EQ-5D Index	0.15**	0.05, 0.24	0.03	−0.07, 0.13

**p < 0.01

### Criterion validity

To examine criterion validity, the two subscales were evaluated in relation to PAM-13 score and health confidence score. The Spearman correlation results showed that Commitment and Navigation scores were moderately correlated with PAM-13 score (for Commitment: rho = 0.55, 95% CI = 0.48, 0.62; for Navigation: rho = 0.48, 95% CI = 0.40, 0.55) and health confidence score (for Commitment: rho = 0.47, 95% CI = 0.39, 0.54; for Navigation: rho = 0.35, 95% CI = 0.26, 0.43).

The comparison of the two subscale scores across PAM activation levels and between high and low health confidence groups (Table 5) showed that Commitment and Navigation scores increased with the increase of PAM and health confidence levels (all *p* < 0.001). Dunn’s test results showed that there was a significant difference in Commitment and Navigation scores between any two PAM levels among PAM level 2 to level 4. The insignificant score difference between level 1 and level 2 could probably be explained by the small number of participants with PAM level 1 (*n* = 8).

**Table 5:** The mean subscale scores of the 8-item ACE Measure by PAM activation and health confidence levels (*N* = 400)

Levels	n	Commitment (range: 0 – 25)		Navigation (range: 0 – 25)	
		Mean	SD	Mean	SD
PAM activation level					
Level 1	8	13.9	5.3	12.9	3.7
Level 2	41	15.6	3.5	15.1	2.5
Level 3	281	18.3**	2.6	17.0**	2.9
Level 4	70	21.3**	2.5	19.9**	3.2
Health confidence level					
Low	46	15.5	3.9	15.5	2.8
High	354	18.8**	2.9	17.5**	3.2

**p < 0.01 compared to the first category using Dunn’s tests

## Discussion and conclusions

### Discussion

The present study examined the psychometric properties of the 8-item ACE Measure among English-speaking community-dwelling adults in Singapore. The CFA results confirmed that the two-factor structure (Commitment and Navigation) fit the data well with some model modifications. The considerably high factor loadings demonstrated simple structure of each subscale and indicated good factorial validity – one of the four types of evidence for construct validity [38]. The analysis of floor and ceiling effects for individual items revealed that “strongly disagree” was very rarely selected (0 – 2.3%) and the percentages of “strongly agree” ranged from 8.5 to 22.5%, with half of the items presenting ceiling effects. However, there were no floor and ceiling effects for each subscale of the 8-item ACE Measure.

The internal consistency reliability results were good for Commitment. The item-rest correlations for Navigation subscale were lower than 0.50 for all four items and the reliability coefficient was lower than 0.70, suggesting the internal consistency reliability of the Navigation subscale is questionable. However, the unsatisfactory internal consistency of the Navigation subscale was also pronounced in the previous validation studies (23,24), indicating the issue might lie with the original items of Navigation. Items like “I feel comfortable talking to my doctor about my health.” and “Different doctors give different advice; it’s up to me to choose what’s right for me.” had relatively lower factor loadings. This could be due to patient’s comfort level in being able to talk to the doctor about health or a person’s attitude towards doctors’ advice may be influenced by many other factors (e.g. personality, trust or doctor-patient relationship, and cultural beliefs) [39] other than one’s confidence and ability to ask about and participate in treatment decisions. This indicates that the items under Navigation needs for further improvement by either revising the existing questions or including additional items.

Comparison of the Commitment and Navigation scores across individuals with different self-perceived health status showed that those with perceived health not as good as peers tended to have lower Commitment score than those who perceived their health as good as or better than peers, which is consistent with our hypothesis and the results of the original ACE validation study [23]. Furthermore, consistent with findings reported by studies using other patient engagement measures [11, 40, 41], those with poorer health-related outcomes, i.e. lower frequency of activity participation and poorer HRQoL demonstrated being less engaged. Although the study cannot infer causal relationships between engagement and health-related outcomes based on the nature of cross-sectional design, the associations serve as hypothesis-testing validity, and thus support the construct validity. Our hypothesis of a significant relationship between education levels and Navigation scores was supported by the data, which further supports the hypothesis-testing validity. Although those with higher education levels also had higher Commitment scores, the association was not significant.

The moderate associations between each subscale and the scores of the well-established PAM-13 and Health Confidence Measure, and the linear trends of the two subscale scores across PAM levels support the criterion validity.

It is worth mentioning that different from the ACE-12 Measure, the 8-item ACE Measure only have two subscales (Commitment and Navigation). In Singapore, primary healthcare system includes government polyclinics and private general medical practitioner (GP) clinics. There is no official primary care doctors’ information available online at national wide and residents seeking treatment at polyclinics are not able to select a particular doctor as the next available doctor will be randomly allocated to them. Although GP clinics offer higher selection feasibility, the higher cost of care and lack of comprehensive facilities (e.g. onsite laboratory and imaging services) result in most residents, especially chronic disease patients, seeking treatment mainly from government polyclinics [42, 43]. With these characteristics of primary healthcare system, residents in Singapore are less prone to choose doctors or look for information online when choosing a new doctor, especially older residents. Given that Informed Choice should be an essential component of patient engagement [23], it is necessary to develop items relevant to the Singapore context. Instead of capturing choosing a new doctor for care, capturing awareness of options to choose from several diagnostic tests or treatments, knowing the details, benefits, risks and expected outcome of each for informed choice might be more appropriate. Further research on selecting relevant items to measure this dimension would be useful in making the patient engagement measure more holistic.

With the current shift of health care from hospital to community in Singapore and given the relationship between patient engagement and improved health outcomes, it is important to better understand and measure patient engagement among our population. A validated measure will enable us to examine how a patient is ready to take care of their own health in the community and to evaluate the effectiveness of health coaching programs on patient activation or engagement. It will also allow for better intervention recommendation and health resources allocation.

### Conclusions

The two-subscale ACE Measure demonstrated good factorial validity and criterion validity. It also had moderate correlation with frequency of activity participation in the study population. However, evidence in internal consistency was mixed with unsatisfactory Cronbach alpha for Navigation, indicating a need for improvement for Navigation subscale. Further research incorporating items reflecting informed choice which are relevant to the Singapore context is needed to make the measure more holistic.

## Data Availability

According to the Data Protection Act Commission Singapore-Advisory Guidelines for the Healthcare Sector, all the individual data collected for the Population Health Index study are protected under the Act. As such, the datasets analyzed during the current study are not publicly available. However, minimal dataset underlying the findings in the manuscript is available from the corresponding author on reasonable request.

## Abbreviations

ACE Measure: Altarum Consumer Engagement Measure™
CFA: Confirmatory Factor Analysis
CFI: Comparative Fit Index
CI: Confidence interval
GP: General medical practitioner
HRQoL: Health-related quality of life
PAM: Patient Activation Measure
PHE: Patient Health Engagement
PHI: Population Health Index
SD: Standard deviation
TLI: Tucker-Lewis Fit Index
RMSEA: Root Mean Square Error of Approximation
VAS: Visual Analog Scale
